# Mr. Kiernan on the Anatomy and Physiology of the Liver

**Published:** 1834-04-01

**Authors:** 


The Anatomy and Physiology of the Liver. By *Francis Kiernan*. Esq. Member of the Royal College of Surgeons, late Teacher of Anatomy. London, 4to, pp. 60, 4 Copper Plates. 1833.

[From the Philosophical Transactions.]

The progress which has lately been made on the Continent, in the departments of anatomy, physiology, and pathology, should give rise to serious reflections in the minds of the scientific members of the medical profession in Britain. It may not be flattering to our national vanity or pride, indeed we should grieve if they were not seriously mortified, when we consider that our best modern works on descriptive anatomy, and most of our advances in the anatomy of disease, are borrowed or are stolen from the Germans and the French.

The fact is too true—the explanation is not difficult. We are not deficient in invention—in steady industry we are unequalled—necessity presses too hardly on many of us—why, then, are we beaten by the foreigners, in the most exact and most philosophical province of medicine? The reason, alas, is obvious and humiliating. Money is a secondary object abroad—it is the alpha and omega of this land. However high-minded a man may be—however he may smile at fashionable folly, and scorn the prostration of the vulgar before aristocracy and wealth, he is soon made to feel that science and professional enthusiasm are sorry defences against the real evils, and as real contumely, that poverty entails upon her followers here. If there ever was a country in which want of property was looked on as a crime, that country is England now. The philosopher out at elbows, shunned by society, famished by want, finds no alleviation of his actual hardships in the kind and specious consideration of the world.

How different it is abroad, those who are acquainted with foreign manners can amply tell. The littérateur there lives upon a little—mixes with society—is treated with respect—and forms a portion of a scientific côterie, possessed of spirit, and energy, and influence. True, there is *the* exclusive class in Germany and France. But it is a class, and a small one too, and the man of science does not need, and does not pine for, its entré. In England, the exclusives are of no special breed—they permeate all ranks—jaundice all eyes—and the spirit of aristocracy is the spirit of wealth. The poor of all trades and all professions are despised—they are a Pariah caste.

What wonder, then, that we struggle to make money. Mammon is our household god, the first of our Di Penates. Without him, we possess no honour, no happiness. He *must* be worshipped.

Our neighbours have made provision for science in their numerous professorships. The moderate salary secures the professor the means of living, and, through the constitution of society, ensures his admission to the circles of his equals or superiors. Wealth is not necessary to distinction—perhaps it is almost unattainable. Ambition, which is ever active, points out the road to fame, and its effects are visible in the actual condition of medicine.

It is singular that Adam Smith objected to endowments, for the advancement and encouragement of learning. He applied to science the political principle of supply and demand. This question is considered with some ability in a recent Number of the Edinburgh Review.* We will take the liberty of selecting one passage; it is a comment on the reasoning of Adam Smith, and follows a satisfactory answer to Turgot.

"The objection of Smith takes broader ground. He applies the principle of supply and demand (so conclusive in the facts with which the science of political economy is concerned) to our moral and intellectual nature. Wherefore, it is said, give bounties in the shape of endowments, and so pay beforehand for a thing, which, if it is worth having, will pay itself? The principle proscribes private, as well as state endowments; and even the help of voluntary subscription, as either superfluous, or false encouragements. How wofully far this is from being a correct picture of the appetence of mankind for moral, and religious, and scientific truths, is, alas! a matter of daily and melancholy experience. Every body is agreed that it is one of our first duties—but those who are best entitled to speak, are well aware, it is also one of our greatest difficulties—to create and accelerate this demand. The question is a question of fact, concerning human nature. May these things be left to find their level? or, unless a supply is forced, so as to be beforehand with the demand, is it not too probable that there will be no demand at all? On this point, Dr. Chalmers, in his very able, and not sufficiently known tract upon *Endowments*, appears to have left nothing essential to be added. A single exception, admitted upon principle, is fatal to the axiom on which Dr. Smith has grounded his proposition. He has himself tendered in this exception, by requiring that Schools should be provided for the *lower orders*. To recommend that food for the *mind* should be thus supplied them, and to insist that they may be trusted to procure food for the *body* for themselves, is to concede at once the true distinction between the two cases. But the distinction is not peculiar to the lower orders and to elementary learning. The rich are quite as averse as the poor to listen to, or to remunerate their instructors. Ask of the booksellers the market price of science. Ascending upwards, subjects of the mightiest import to nations and to mankind, would never remunerate their cultivators with bread and cheese. France and Germany, where literature is a great deal thought of, and riches very little, are quite aware of this. Men like Hooker, Jeremy Taylor, and Locke, if left to the profit they could make as tradesmen by the sale of knowledge, would scarcely get the wages of an expert mechanic. It is a fact which experiments enough have verified—no thanks to us—that knowledge has a reward of its own incommensurate with money." 496.

We need not state our reasons for concluding, that society is undergoing a perceptible revolution in this country. We are gradually approximating to the Continental standard. On some accounts this is well—on many it is not. Come what come may, we do hope that more encouragement will be held out to abstract science than has hitherto been offered. Endowments and professorships in medicine are wanted—especially in those departments where labour, without them, brings no reward, or none of that description which is indispensable for ease and comfort.

We have, not unnaturally, been led to these reflections by a glance at the work before us. It is almost a phenomenon—a laborious work upon anatomy by a gentleman in general practice.

Such attempts neither do nor can receive much public patronage or favour.

* Edinburgh Review, January, 1834.

It is the duty of the profession to extend its hand in aid of inquiries like this. Mr. Swan is an example of what, under the present constitution of the profession, the laborious cultivator of anatomy may look for. That gentleman has been engaged for seven or eight years in the prosecution of his dissections and the preparation of his drawings. It cannot be doubted that they do credit to this country. Yet we venture to affirm that Mr. Swan has incurred much pecuniary loss, and we fear there is little prospect of an ultimate adequate return.

It has formed a subject of complaint that our colleges do not patronize labours of this description. They cannot. They may offer indeed to defray the expense of publication, as we hear that the College of Surgeons did in the case of Mr. Swan. But this is no recompense for years of toil, and the man who could afford to reject the paltry assistance, would feel humiliated at receiving the charitable dole.

We repeat that there should be in our profession the means of rewarding in an honourable manner the aspirant for purely scientific distinction. There is an ample field in anatomy, physiology, and pathology for young men of industry, talent, and zeal. But their ardour is soon chilled by the comfortless prospect that field displays. Years of toil are in the foreground—neglect and poverty occupy the horizon. They turn with dismay from the hopeless enterprize, and seek the means of subsistence and of fortune in intrigues for private connexion, and in cunning or desperate attempts to arrest the attention of the public.

The enormous taxes wrung from Englishmen are inadequate, it would appear, to enable our Government to do that for science which the needy and despotic authorities of the Continent have done. The philosopher may console himself with the flattering reflection, that the parasite of the minister and minion of the court have better claims than he upon the public purse. Whether a change in the feelings of the community and determinations of the legislature may be reasonably anticipated, is more than we shall take upon ourselves to prophesy. We trust that so soon as the constitution of our own corporations has assumed a form of consistency and durability, this subject will attract their serious attention. We shall probably return to it when fitting opportunities present themselves.

We pass to the consideration of the work of Mr. Kiernan. The nature of the subject and the character of the description preclude a strictly analytical notice.

Mr. Kiernan adopts the following arrangement in his description of the liver:—first, he considers the lobules, their disposition, their connexions with each other and with the vessels; secondly, the surfaces of the liver and the distribution of the vessels; and thirdly, the structure of the lobules. We shall, in some degree, confine ourselves to the parts which are most striking, or most important.

### Of the Lobules.

"The hepatic veins with the lobules present a tolerably accurate resemblance to the trunk, branches and leaves of a tree. The lobules may be compared to the leaves. The substance of the lobules is arranged around the minute branches of the veins in a manner which may be compared to the disposition of the parenchyma of a leaf around its fibres. The vessels in which the minute veins terminate may be compared to the branches of the tree, and these vessels by their junction form the trunks. The hepatic veins may be divided into two classes; into those veins contained in the lobules, and those contained in canals formed by the lobules. The first class is composed of the intralobular branches, one of which occupies the centre of each lobule, and receives the blood from a plexus formed in the lobule by the portal vein. The second class of hepatic veins is composed of all those vessels contained in canals formed by the lobules. Numerous small branches, as well as the large trunks which terminate in the inferior cava, are included in this class; they all resemble each other in being contained in canals, and they differ from the vessels of the first class which are contained in the lobules. The intralobular veins terminate in some of these vessels, and not in others; these vessels therefore admit of being divided into two sets; 1st, those in which the intralobular branches terminate; 2nd, those in which no intralobular branches terminate. The lobules are arranged around the veins composing the first set, the bases of these bodies resting upon them; they may be called the sublobular hepatic veins, this term being applied to them merely to distinguish them from the trunks which compose the second set, and on which the bases of the lobules do not rest. The branches of the second set are formed by the junction of those of the first; the canals containing the former differ in the manner of their formation from those containing the latter. Every branch of the hepatic veins contained in the liver belongs to one of these two classes of vessels.

Each intralobular vein is composed of a central vessel, and of from four to six or eight smaller vessels, which terminate in the central vessel. The intralobular veins invariably correspond in form with the lobules, the substance of which is arranged around them; and as these vessels resemble in some degree the fibres of a leaf, so sections of the lobules made in the direction of the vessels assume a more or less foliated appearance. The lobules are not, however, flattened bodies like leaves; for, as the smaller veins enter the central vein in every direction, so small processes project in every directien from the lobules, the number of processes being equal to the number of veins terminating in the central vein. The form of the lobules will be now easily understood; their dimensions are known to all anatomists. They are small bodies, arranged in close contact around the sublobular-hepatic veins, each presenting two surfaces. One surface of every lobule, which may be called its base, rests upon a sublobular vein, to which it is connected by the intralobular vein running through its centre, the base of the lobule thus entering into the formation of the canal in which the sublobular vein is contained. The canals containing the hepatic veins may be called the hepatic-venous canals or surfaces; and as the base of every lobule rests on a sublobular vein, it its evident that the canals containing these veins are formed by the bases of all the lobules of the liver. The external or capsular surface of every lobule is covered by an expansion of Glisson's capsule, by which it is connected to, and separated from, the contiguous lobules, and in which branches of the hepatic duct, portal vein and hepatic artery ramify. All the lobules resemble each other in their general form, and they are all of nearly equal dimensions; they appear larger when the section is made in the direction of the hepatic veins, and smaller when in the transverse direction. This is most apparent in that state of the liver usually called the nutmeg liver. In a longitudinal section of a lobule, the intralobular vein is seen running through its centre; and if on the surface of the section five of the projecting processes of the lobule be seen, five smaller veins will also be seen, one occupying the centre of each process, and all terminating in the central vein. In a transverse section of a lobule, the divided extremity of the intralobular vein is seen in the centre, and three or four processes of the lobule are seen shooting out in different directions. The vein being thus always situated in the centre, it sometimes happens that on the surface of a section of the liver, veins are seen in some lobules and not in others; this appearance is caused by the instrument, which, passing obliquely through these lobules, divides some vessels, which thus become apparent, and passes either above or below others.

The superficial differ in one respect from the internal lobules. In the latter, the intralobular veins commence at a certain distance from the surfaces of these bodies, the substance of which completely surrounds them, except at the bases of the lobules, where the veins make their exit to terminate in the sublobular veins. By superficial lobules are meant, not those only which form the convex and concave surfaces, but those also the capsular surfaces of which form the canals containing certain branches of the hepatic duct, portal vein, and hepatic artery, and the canals containing the trunks of the hepatic veins, all these canals being tubular inflections inwards of the superficies of the liver. In all the superficial lobules, the intralobular veins commence immediately at the surfaces; these lobules appearing less perfect in form, or less developed, than those of the interior, or as if their upper portions had been removed, giving to the surfaces of the organ the appearance of the surface of a section." 715.

In alluding to the opinions of Mascagni, Mr. Kiernan makes the following remarks:

"Mascagni, adopting Malpighi's view of the arrangement of the lobules, compares the liver to a bunch of grapes; and this anatomist and Bidloo have represented the lobules appended to the extremities of the vena portæ. As certain branches of this vein first ramify between the lobules, and finally enter them, these bodies may be represented as appended to its extremities: and although every lobule receives branches from this vein, yet a certain number only are clustered around its trunks, with which they have no immediate connexion; whereas the base of every lobule in the liver is in contact with, and connected to, an hepatic vein.

The essential part of a gland is undoubtedly its duct; vessels it possesses in common with every other organ; and it may be thought that in the above description too much importance is attached to the hepatic veins: but relations similar to those which exist between these veins and the lobules, do not exist between the latter and the ducts, or between them and any other set of vessels; nor is there the same exact relation between the ducts and lobules as between these bodies and the hepatic veins, for a lobule with six projecting processes may have three times that number of ducts ramifying on its external surface, whereas the same lobule will have but six minute veins, one in each process, all of which terminate in the central intralobular vein." 716.

Passing over the account of the *external surface* of the liver, we may pause for an instant at that of the PORTAL CANALS.

These commence at the transverse fissure, where they are continuous with the concave surface of the liver; they contain the hepatic ducts, the portal veins, the hepatic arteries, and the vaginal branches of all these vessels, with the nerves and absorbents, enveloped in a sheath of cellular tissue, first described by Glisson, and called Glisson's Capsule. These canals, and those containing the large hepatic trunks, are formed by the capsular surfaces of a limited number of lobules; the canals containing the sublobular-hepatic veins are formed by the bases of all the lobules. As the portal vein is the largest vessel contained in them, they may be termed the portal canals.

Glisson's capsule is to the liver what the pia mater is to the brain; a cellulo-vascular membrane, in which the vessels divide and subdivide to an extreme degree of minuteness; which lines the portal canals, forming sheaths for the larger vessels contained in them, and a web in which the smaller vessels ramify; which enters the interlobular fissures, and, with the vessels, forms the capsules of the lobules; and which finally enters the lobules, and, with the blood-vessels, expands itself over the secreting biliary ducts. Hence arises a natural division of the capsule into three portions, a vaginal, an interlobular, and a lobular portion; and as the vessels ramify in the capsule, their branches admit of a similar division.

At the transverse fissure the hepatic duct, vena portæ, and hepatic artery divide into branches which enter the portal canals. Of these canals the ultimate ramifications, however small, contain each a branch of each of these vessels. In the lobules the ducts form plexuses, as do the veins and arteries; the latter are exceedingly minute and few in number: they are the nutrient vessels of the lobules, and probably terminate in the plexuses formed by the portal vein.

Mr. Kiernan next dwells on the vaginal portion of Glisson's capsule and its vessels. After a minute description he observes that, it is evident that Glisson's capsule is a cellulo-vascular membrane, composed of the vaginal branches of the duct, vein and artery, ramifying in a layer of cellular tissue. Its existence around the three vessels in the larger canals, in which the vaginal plexus is most complicated; its existence on that side only of the smaller canals occupied by the duct and artery, and its almost total absence on the opposite side, sufficiently prove that by its means the three vessels are brought into apposition with all the interlobular spaces on the surfaces of the canals.

The following extract may be added.

"The coats of the ducts are highly vascular; the rugæ on their internal surface, and those on the internal surface of the gall-bladder, are formed by the ramifications of the larger blood-vessels, arteries as well as veins, covered by the mucous membrane. This membrane is studded with vascular papillæ, which become remarkably developed in the diseased ducts so frequently found in sheep and oxen. The smaller ducts are furnished with papillæ only, and to the rupture of the delicate vessels forming these papillæ is to be attributed the facility with which Sœmmerring and other anatomists injected the ducts from the arteries and veins, and not to any direct communication between the vessels and the ducts. This point has been particularly insisted upon by Muller, who, in speaking of Walter's experiments, says, 'Itaque si in Walteri experimentis massa interdum ex vasis sanguiferis in ductum hepaticum transiit, certe non per minimos ductus biliferos transiit, sed in truncos ipsos ex vasculis sanguiferis erupit.' Mappes imagines that the hepatic artery is principally destined to supply the coats of the portal vein with blood: this is so far from being the case, that when the arteries are well injected, the larger ducts, from the extreme vascularity of their coats, may be mistaken for the injected arteries, whilst, in the coats of the vein, no vessels will be detected without the aid of the magnifying glass.* The coats of the ducts may be as highly injected from the portal vein as from the hepatic artery; but they cannot be injected from the hepatic veins, if the injection is confined to these vessels, and does not return by the

* "Mappes probably saw the vaginal arteries, which ramify on the parietes of the canal previously to entering the interlobular spaces, through the transparent coats of the veins, and concluded that they were ramifying in the coats of these vessels; or in making sections, this anatomist may have removed a portion of the parietes of a canal, leaving the arteries on the vein."

portal vein. Mappes could not inject the ducts from the portal or hepatic vein; he is nevertheless of opinion, 'que la bile est tirée plutôt du sang déjà parvenu dans cette veine (la veine hépatique) que de celui qui se trouve encore dans les dernières extrémités de la veine porte.' The ducts cannot be injected in a direct manner from the hepatic vein, no branches of this vein ramifying in their coats; fluid may indeed be made to pass from this vein into the ducts, but only through the medium of those branches of the portal vein which ramify in the coats of the ducts.* The ducts are injected from the portal vein and from the hepatic artery in the same manner as the fœtal intestine is frequently filled with injection from the umbilical vein or aorta, viz. by the rupture of the minute vessels of the mucous membrane. Hence it is evident that the ducts, so far as they have been yet traced, are abundantly supplied with arterial blood; that this blood returns into the branches of the portal, and not into those of the hepatic veins; and that the hepatic portal vein has branches of origin in the coats of the excreting ducts from the terminations of the hepatic artery, as the abdominal portal vein arises in the coats of the intestines, in the spleen and pancreas, from the arteries of these organs." 727.

In speaking of the *inter-lobular portion of Glisson's capsule and of its vessels*, Mr. Kiernan observes that all the branches of the portal vein communicate with each other through the medium of the interlobular branches. This statement is opposed to the assertions of Bichat and Mappes.

### Of the Hepatic Veins and Hepatic Venous Canals.

The hepatic veins, says our author, are contained in canals, which may be called the hepatic venous canals; they commence in the interior of the liver, and terminate at the fissure of the inferior cava. Those containing the hepatic trunks are formed by the capsular surfaces of a limited number of lobules; those containing the sublobular-hepatic veins, are formed by the basis of all the lobules.

Mr. Kiernan makes a remark on Glisson's capsule to which we may allude. He maintains that its structure and uses are fully explained, it being evident that the loose connexion of the ducts, portal veins, and hepatic arteries to the substance of the liver arises from the circumstance of the three vessels ramifying in the same canals; and that the adhesion of the hepatic veins to the substance depends on one vessel only being contained in each hepatic venous canal.

Omitting much minute description we may pause at the following contrast of the portal and hepatic veins.

"By contrasting the hepatic veins with the portal vein, we find that no two intralobular branches of the former anastomose with each other; that the interlobular branches of the latter form one continuous plexus throughout the whole liver; that the sublobular veins anastomose directly, and not through the

---

* "By examining the surface of the liver after injecting the hepatic veins, we may ascertain in which parts the coats of the ducts are injected from these vessels, and in which they are not. If on one portion of the surface we see the interlobular portal veins injected from the hepatic veins, a few injected vessels will be found in the coats of the ducts of this part. In other parts of the liver, where the injection is confined to the centres of the lobules, and consequently to the hepatic veins, no injection will be found in the coats of the ducts, although the injected hepatic veins will be seen through them."

medium of the intralobular branches; that the portal veins have no direct communication with each other, but anastomose by means of their interlobular branches; that the hepatic veins, like the other veins of the body, proceed in a direct course to their termination in the cava; that the portal vein, accompanied by an artery, resembles an artery in its ramifications; that the larger hepatic veins, having longitudinal fibres in their coats, differ in structure from the portal vein; and that the blood contained in the liver after death is almost invariably found in the hepatic veins, the portal vein being usually empty." 737.

### Of the Structure of the Lobules.

After noticing once more the opinions of Ruysch, Malpighi, and others, Mr. Kiernan affirms that to Muller is due the important discovery, that a gland is a duct, with bloodvessels ramifying on its parietes.

The portal vein enters the liver in all vertebrated animals, in all of which the lobules are arranged around the hepatic veins. Each lobule is composed of a plexus of biliary ducts, of a venous plexus formed by branches of the portal vein, of a branch of an hepatic vein, and of minute arteries. Nerves and absorbents must be presumed to enter them.

The hepatic ducts, commonly so called, and their vaginal and interlobular branches constitute the excreting portion of the biliary apparatus; they are also organs of mucous secretion, being furnished with mucous follicles: the secreting portion of the liver is also composed of ducts, which form a plexus in each lobule. These plexuses may be called the lobular biliary, or secreting biliary plexuses.

"Examined with the microscope, the injected interlobular ducts are seen dividing into branches, which, entering the lobules, divide and subdivide into minute ducts; these ducts anastomose with each other, forming a reticulated plexus. If an uninjected lobule be examined and contrasted with an injected lobule, it will be found that the acini of Malpighi in the former are identical with the injected lobular biliary plexus in the latter, and the bloodvessels in both will be easily distinguished from the ducts. The ducts forming the plexuses, when examined with the microscope, present very much the appearance of cells; and this appearance, which has been well delineated by Mascagni, probably induced this anatomist to consider the liver as an assemblage of minute cavities, giving origin to the ducts. The form of the lobules bears no relation to the arrangement of the ducts, the form of each lobule being always correspondent to the branches of the intralobular hepatic vein occupying the centre of the lobule. The coats of the lobular ducts, on which the blood-vessels next to be described ramify, constitute the proper secreting substance of the liver, as the coats of the cortical ducts of the kidney, and those of the tubuli seminiferi, constitute the secreting substance of their respective organs." 742.

In describing the *lobular venous plexuses,* Mr. Kiernan mentions in a more distinct manner than before the communication between the portal and hepatic veins. The interlobular branches of the portal vein, surrounding the lobules on every side except at their bases, divide into branches which, entering these bodies, form in each of them a plexus, the branches of which terminate in the intralobular hepatic veins situated in the centre of the lobule. This plexus interposed between the interlobular portal veins and the intralobular hepatic vein, constitutes the venous part of the lobule, and may be called the lobular venous plexus. The venous plexus of one lobule communicates with the plexuses of the surrounding lobules by means of the intervening interlobular branches of the vena portæ, this vein thus forming one continuous plexus through the whole liver. The converging branches of each plexus unite at the centre of each lobule, and form an intralobular hepatic vein, this vein having no communication with the corresponding veins of the contiguous lobules, except through the medium of the intervening plexus and portal veins. No branches of the hepatic veins are found in any other part of the liver; occupying the centre alone of each lobule, their only office is to convey the blood from the lobular venous plexuses, and not from the arteries.

"The venous plexus ramifies on the biliary plexus; the blood circulating through it is composed of the portal blood, and certainly of that portion of the arterial blood which, having nourished the excreting ducts and supplied them with mucus, and having circulated through the vasa vasorum of all the vessels, becomes venous and is received into the branches of the portal vein, by which, with the portal blood, it is conveyed to the plexus; and from this mixed blood the bile is secreted." 746.

After describing some experiments on the injection of the lobular arteries, Mr. Kiernan infers from their result, that the secreting portion of the liver, like the excreting portion of the kidney, is supplied with arterial blood for nutrition only. As all the branches of the artery, the termination of which can be ascertained, end in branches of the portal vein, he thinks it probable that the lobular arteries terminate in the lobular venous plexuses formed by that vein, and not in the intralobular branches of the hepatic veins, which cannot be injected from the artery, the blood of these arteries, after having nourished the lobules, becoming venous, and thus contributing to the secretion of the bile. In short, Mr. Kiernan asserts that he has shewn, that no branches of the hepatic artery terminate in the hepatic veins, the latter vessels being injected from the former only through the medium of the lobular venous plexuses of the portal vein.

### Of the Red and Yellow Substances of the Liver.

These, which have been described as separate and distinct by Ferrein, Mr. Kiernan affirms to be one and the same; for the structure of the lobules is similar, and that of each is homogeneous. Neither is one part more vascular than another.

We extract an anatomical account of the modes in which congestion of the liver occurs.

" Sanguineous congestion of the liver is either general or partial. In general congestion the whole liver is of a red colour, but the central portions of the lobules are usually of a deeper hue than the marginal portions. Partial congestion is of two kinds, hepatic-venous and portal-venous congestion. Of the first kind there are two stages. In the first and most common stage, the hepatic veins, their intralobular branches and the central portions of the plexuses are congested. The congested substance is in small isolated patches of a red colour, and, occupying the centres of the lobules, it is medullary; the non-congested substance is of a yellowish white, yellow or greenish colour, according to the quantity and quality of the bile it contains: it is continuous throughout the liver, and, forming the marginal portions of the lobules, is cortical. This is passive congestion of the liver; it is the usual and natural state of the organ after death, and probably arises from its double venous circulation. In the second stage, the congestion extends through the plexuses to those branches of the portal vein situated in the interlobular fissures, but not to those in the spaces, which being larger than, and giving origin to, those in the fissures, are the last to be congested; when these vessels contain blood, the congestion is general, and the whole liver is red. In this second stage, the non-congested substance appears in isolated circular and ramous patches, in the centres of which the spaces and fissures are seen. This is active congestion of the liver; it very commonly attends disease of the heart, and acute disease of the lungs or pleuræ: the liver is larger than usual, in consequence of the quantity of blood it contains, and is frequently at the same time in a state of biliary congestion, which probably arises from the sanguineous congestion. Although in the first stage, the central portions of the plexuses, and in the second, the greater portion of each plexus, and those branches of the portal vein occupying the fissures, are congested, and although the plexuses are formed by the portal vein; yet, as this form of congestion commences in the hepatic veins, and extends towards the portal vein, and as it is necessary to distinguish this form from that commencing in the portal vein, the term of hepatic-venous congestion will not probably be deemed inapplicable to it. Portal-venous congestion is of very rare occurrence; I have seen it in children only. In this form the congested substance never assumes the deep red colour which characterizes hepatic-venous congestion; the interlobular fissures and spaces, and the marginal portions of the lobules, are of a deeper colour than usual; the congested substance is continuous and cortical, the non-congested substance being medullary, and occupying the centres of the globules. The second stage of hepatic-venous congestion, in which the congested substance appears, but is not cortical, may be easily confounded with portal venous congestion." 754.

Mr. Kiernan, having shewn that the liver is not composed of two distinct substances, deems it fair to conclude that it executes only one function—the secretion of bile.

With a summary expression of the facts spread through the preceding pages, we fear we must terminate this lengthened notice.

"It has been shewn that all the vasa vasorum of the liver are branches of the hepatic artery and portal vein; that branches of the portal vein arise in the coats of the hepatic veins themselves; and that the veins of the coats of the vessels constitute the hepatic origin of the portal vein. The arterial blood having circulated through the coats of the vessels becomes venous, and is conveyed by the veins arising in the coats of the vessels into those branches of the portal vein which correspond to the vessels in the coats of which the veins arise: thus, from the coats of the vaginal ducts, veins and arteries, they convey the blood into the vaginal veins; and from the coats of the interlobular ducts, veins and arteries, into the interlobular veins. From the coats of the hepatic veins and inferior cava, the blood is conveyed into the interlobular portal veins. In the vaginal and interlobular veins, the blood conveyed from the coats of the vessels becomes mingled with the proper portal blood. This mixed blood is conveyed by the interlobular veins into the lobular venous plexuses, in which the lobular arteries probably terminate, after having nourished the secreting ducts. From the mixed blood circulating through the plexuses, the bile is secreted by the lobular or secreting biliary plexuses.

The blood which enters the liver by the hepatic artery fulfils three functions; it nourishes the liver; it supplies the excreting ducts with mucus; and, having performed these purposes, it becomes venous, enters the branches of the portal vein, and contributes to the secretion of the bile. The portal vein fulfils two functions; it conveys the blood from the artery, and the mixed blood to the coats of the excreting ducts. It has been called the *vena arteriosa*, because it ramifies like an artery, and conveys blood for secretion; but it is an arterial vein in another sense, being a vein to the hepatic artery, and an artery to the hepatic vein. The hepatic veins convey the blood from the lobular venous plexuses into the cava inferior." 756.

Any comment on this Essay would be useless. The laborious investigations on which it is erected display the diligence and zeal of Mr. Kiernan. If these investigations are successful, and the inferences drawn from them correct, the author will justly have earned the honour of settling great and disputed points in anatomy and physiology. We hope, and indeed we trust, that the talent and the perseverance he displays in the intricate field of anatomical discovery, will not be permitted to pass unrewarded. Some means *might* be found of giving an aim, as well as an impulse, to his talents and his taste. We may probably be excused for expressing a desire that Mr. Kiernan may be withdrawn from the drudgery of general practice, to devote himself more exclusively to the prosecution of inquiries connected with the higher provinces of science.

